# Human pluripotent stem cell-derived products: Advances towards robust, scalable and cost-effective manufacturing strategies

**DOI:** 10.1002/biot.201400348

**Published:** 2014-11-19

**Authors:** Michael J Jenkins, Suzanne S Farid

**Affiliations:** Department of Biochemical Engineering, University College London London, UK

**Keywords:** Bioprocess economics, Cell therapy, Regenerative medicine, Scale-up, Pluripotent stem cells

## Abstract

The ability to develop cost-effective, scalable and robust bioprocesses for human pluripotent stem cells (hPSCs) will be key to their commercial success as cell therapies and tools for use in drug screening and disease modelling studies. This review outlines key process economic drivers for hPSCs and progress made on improving the economic and operational feasibility of hPSC bioprocesses. Factors influencing key cost metrics, namely capital investment and cost of goods, for hPSCs are discussed. Step efficiencies particularly for differentiation, media requirements and technology choice are amongst the key process economic drivers identified for hPSCs. Progress made to address these cost drivers in hPSC bioprocessing strategies is discussed. These include improving expansion and differentiation yields in planar and bioreactor technologies, the development of xeno-free media and microcarrier coatings, identification of optimal bioprocess operating conditions to control cell fate and the development of directed differentiation protocols that reduce reliance on expensive morphogens such as growth factors and small molecules. These approaches offer methods to further optimise hPSC bioprocessing in terms of its commercial feasibility.

## 1 Introduction

Human pluripotent stem cells (hPSCs) have opened fresh avenues in modern medicine that have the potential to revolutionise healthcare, particularly since the first derivation of induced pluripotent stem cells (iPSCs) [1, 2]. Human embryonic stem cells (hESCs) and human-induced pluripotent stem cells (hiPSCs) have the capacity to differentiate into all mature cell types, making them attractive candidates for use as cell therapies [3]. Moreover, hPSCs also offer a unique, novel platform by which to augment, and even redefine, current drug discovery and drug screening programmes by the provision of a human in vitro tool on which to perform efficacy and toxicity screens for novel chemical entities (NCEs) [4, 5]. hiPSCs could also pave the way for personalised medicine through the medium of responders versus non-responder ‘trial in a dish’ models [6].

hPSCs can provide a cornerstone of the regenerative medicine industry via the provision of cell therapies for diseases with unmet clinical needs. hiPSCs in particular might provide a diagnostic tool capable of assuaging the high late-phase failure rate of NCEs in clinical trials [7, 8]. The market for stem cell research products exceeded $3bn at the end of 2013 (http://tinyurl.com/n26fe4z). hPSCs for use as research tools are currently marketed at $2000–$3000/vial [9]. However, the value of this market is likely to be incremental when considered against hPSC-derived cell therapies [10], which has the potential to tap into a multi-billion dollar global market [11]. If hPSCs are to achieve their full clinical and commercial potential, significant challenges must be overcome with regards to current abilities to produce hPSC-derived cells at commercially relevant scales.

At the forefront of current challenges to hPSC-derived therapies is the production of cells at a relevant quantity and quality to support their function. Details on the bioprocess techniques for hPSC therapies currently being manufactured for preclinical and clinical trials or for use as research tools are provided in Table 1. To date, hPSC-derived products in development have mainly consisted of retinal progenitor cells and pancreatic β-cells derived from hESCs [12–15], or a variety of cell lineages such as neurons, cardiomyocytes and hepatocytes derived from hPSCs for use as research tools [16]. Promising results have also been observed for cell therapies derived from hiPSCs, such as retinal pigment epithelial cells [17]. Table 2 indicates that most products in clinical development still depend on planar technologies. Traditional, planar technologies that offer reliable tools for laboratory-based protocols are labour-intensive and do not lend themselves to large scale, allogeneic processes [18]. Dose sizes reported for hPSC-derived cell therapy products currently range from 5 × 10^4^ cells for indications such as macular degeneration to 10^8^ cells for diseases such as diabetes (Table 2). Furthermore, it has been estimated that large doses, of around 10^9^ cells, will be needed to treat conditions such as myocardial infarction and liver disease [19, 20]. Whilst this review focuses on process techniques for the production of single-cell type populations, therapies for certain disease types will necessitate transplantation of a functional tissue-like structure. Novel, organoid development techniques may therefore provide a method of production of tissue-like structure representing a variety of different organs from small seed populations of hPSCs. These have potential applications as either transplantable therapies or research tools [21–24].

**Table 1:** Bioprocess development considerations for hPSC-derived products

Consideration	Example Criteria
Operational	Expansion yields (harvest densities)
performance	Expansion folds
	Differentiation efficiencies
	DSP yields
	Purity
	Resource utilisation
	Scalability
	Lot processing time
Economic	Capital investment
	Cost of goods (materials, labour, quality control and indirect)
	Economies of scale – scale-up versus scale-out
	Fresh versus frozen product transportation and storage
	Process development costs
	Supply chain replenishment
	Product shelf-life
	Reimbursement value
Quality control	cGMP and cGTP standards
and regulatory	Process robustness and reproducibility
compliance	Process validation, acceptable ranges of operation
	Product characterisation
	Quality, consistency and source of raw materials
	Automated versus manual processing
Safety	Contamination and containment
	Live human tissue handling
	Patient safety – side-effects, risk of tumour formation
Flexibility	Process changes
	Manufacturing demand changes
	Process bottlenecks
	Process scalability

**Table 2:** Technologies used for expansion and differentiation of hPSC-derived cell products used in clinical trials or as research tools

Company	Indication	Target cell type	Dose size	Cell expansiona) details	Differentiation detailsa)	Source
Cellectis	Diabetes mellitus (type I)	Insulin producing β-cells	ND	SUB: hollow fiber, multicompartment perfusion bioreactor	SUB: hollow fiber, multicompartment perfusion bioreactor	[12]
Advanced Cell Technology	Macular degeneration	Retinal pigment epithelial (RPE) cells	5 × 10^4^	Planar: well-plates, MEF feeder layer, three passages	Planar: well-plates EB formation	[13]
CellCure	Macular degeneration	RPE cells	2 × 10^4^	Planar: well-plates xeno-free media	Planar: well-plates, 8-wk process, serum-free conditions	[14]
Healios	Macular degeneration	RPE cells	5 × 10^4^	Planar: plate-based custom designed automated process platform	Planar: plate-based custom designed automated process platform	[17]
ViaCyte	Diabetes mellitus (types I and II)	Pancreatic β-cell precursors	10^8^	Planar: multi-layer cell factories, 2-wk process, xeno-free media	Planar: plate-based aggregate differentiation, 2-wk process, Xeno-free media	[15]
Geronb)	Spinal cord injuries	Oligodendrocyte progenitor cells	2 × 10^6^	Planar: matrigel coatedT-flasks, 3- to 5-wk process	Planar: T-flasks, 6-wk process, growth factor-based protocol	[111]
CellularDynamics International	hiPSC-derived cells for use as research tools	Cardiomyocytes, neurons, hepatocytes,	N/A	SUB: litre-scale, five passages, Xeno-free media endothelial cells	SUB: litre-scale, chemically defined conditions	[16]

a) Technologies for expansion and differentiation operations are detailed alongside process durations and media where this information is available.

b) Geron's GRNOPC1 therapy was withdrawn from trials but has been included in this table for comparison.

Planar technologies may struggle to satisfy the global demand for hPSC-derived cell therapies requiring high dose sizes [20, 25]. Furthermore, there is widespread use of xenogeneic materials associated with traditional hPSC technologies, preventing their use in the production of clinical grade hPSC-derived cells. Differentiation strategies have often represented idiosyncratic protocols that have proven difficult to translate to robust bioprocess unit operations [26]. Media costs associated with hPSC processes are of further concern; many media supplements render the products of current hPSC processes prohibitively expensive for purpose. Additionally, studies into the poorly understood interactions between hPSCs and the microenvironment provided by media components and cell anchorage materials are only now beginning to take place; significant gaps exist in our knowledge of how the design of such materials affects hPSC activity [27].

Previous reviews in this field have discussed advances in the large-scale expansion and bioreactor-based culture of hPSCs (e.g. [25, 28–30]). Other studies have focused upon the impact of the design of microcarriers and anchorage materials on hPSC bioprocessing (e.g. [27, 31–33]). Herein, we aim to summarise key considerations and methods that might be employed in order to achieve cost-effective bioprocess design across a range of manufacturing scales. Key process economic metrics and drivers are outlined. A discussion of advancements in robust, GMP-based expansion and directed differentiation strategies is provided. Finally, a review of recent innovations in integrated bioprocess design is presented.

## 2 Cell therapy bioprocess economics

When examining process options for hPSC manufacture, it is important to consider not only the operational performance but also the consequences on the economics, quality, regulatory compliance, safety and flexibility. These key considerations are summarised in Table 1. This review pays particular attention to progress made on improving the economic and operational feasibility of hPSC bioprocessing.

Reimbursement pressures have resulted in an increased awareness of the importance of estimating and improving manufacturing costs for stem cell products. This section discusses factors that influence two key cost metrics: fixed capital investment (FCI) and cost of goods (COG).

### 2.1 Capital investment

The FCI represents the cost to build a manufacturing facility ready for start-up. It includes the cost of the building with the fixed (non-disposable) equipment, piping, instrumentation and utilities installed. Estimates of facility costs are often made using factorial estimates. These are well established for traditional stainless steel biopharmaceutical facilities using the Lang Factor method [34], which involves multiplying the total equipment purchase cost by the ‘Lang factor’. At present, there are no published studies that have determined an appropriate factorial method for stem cell manufacturing facilities. The Lang factor is usually derived based on the analysis of costs of previous projects; as yet very few FCI benchmarks have been published for stem cell manufacturing facilities. Investment costs for stem cell facilities will also be influenced by the degree of open versus closed processing and the consequences on the cleanroom classification required and whether automated or manual process techniques are employed. Stem cell bioprocessing is also dependent on disposable or single-use process platforms such as T-flasks, CellStacks and single-use bioreactors (SUBs). To this end, a Lang factor method adapted for disposable-based biopharmaceutical facilities is currently the best method available by which to approximate the FCI associated with stem cell manufacturing [35]. Ongoing work at University College London is focused upon developing a method for estimating FCI that is specific to stem cell manufacturing facilities.

### 2.2 Cost of goods

The COG represents the cost to manufacture a stem cell product and comprises direct (e.g. materials) and indirect (e.g. maintenance) costs. Simaria et al. [20] summarise key factors that influence COG values for stem cell products; these include process efficiencies (e.g. harvest densities post-expansion, differentiation yields), technology choices (e.g. planar vs. microcarrier-based SUBs), and resources required and their unit costs (e.g. media, single-use vessels and labour). Economies of scale are a relevant factor as demand and lot size are varied as well as dose for cell therapies and required cell population sizes for cells as drug screening tools. Outputs are usually expressed as COG per cell population for screening tools or COG per dose for therapeutic applications.

Decision-support software can aid the design of cost-effective bioprocesses. However, to date, few published cost studies exist for stem cell bioprocessing. Commercially available flowsheeting software packages have been employed to cost stem cell process designs at fixed scales [36]. Simaria et al. [20] and Hassan et al. [37] present the development and application of decisional tools that integrate models for mass balancing, equipment sizing and bioprocess economics with optimisation algorithms for allogeneic mesenchymal stem cell (MSC) production. The tools were used to predict the most cost-effective upstream and downstream technologies for commercial MSC manufacture across a range of different scales and doses. The analyses presented in these works illustrate how such tools can be used to determine the scale at which planar technologies cease to be cost-effective in contrast to microcarrier-based SUBs, when downstream processing bottlenecks occur, as well as future required performance capabilities of promising technologies to close existing technology gaps and meet COG targets. This approach is being extended [38] to hPSC processes, which typically have additional process steps such as differentiation, so as to identify key economic drivers for drug screening and therapeutic applications, and predict technical innovations required to bridge the gaps constraining widespread application of hPSCs.

In addition to considering the costs of stem cell bioprocessing alternatives, it is also useful to capture the impact of uncertainties such as lot-to-lot variability and contamination risks, particularly when manual processing techniques are employed [39]. Stochastic modelling techniques, such as the Monte Carlo simulation method, have been used to evaluate process robustness under uncertainty in the biopharmaceutical sector [35, 40, 41]. Stochastic modelling has yet to be applied to iPSC processing, but will form an important part of future work in order to develop robust and cost-effective hPSC bioprocesses.

### 2.3 Process economic drivers

In order to achieve cost-effective bioprocesses for hPSCs, efforts need to focus on increasing the overall productivity and/or decreasing the overall production costs. Hence, critical process economic drivers for hPSC processes include expansion and differentiation yields as well as the cost of materials and labour. The remainder of this paper therefore focuses on progress made on the expansion and differentiation yields in planar and bioreactor technologies as well as the development of media and cell-anchorage materials for these unit operations.

## 3 hPSC bioprocessing strategies: Expansion

### 3.1 Planar culture systems for hPSC expansion

Until recently, planar hPSC culture platforms relied heavily on the use of xenogeneic growth substrates and non-human feeder layers, which risk contamination of hPSC-derived products. Furthermore, feeder layers differentially secrete signalling factors, resulting in poorly defined culture conditions that are unsuitable for empirical study of hPSC expansion [42]. The development of anchorage materials comprising of a mixture of synthetic and/or recombinant biological motifs have allowed hPSC culture to progress away from the use of feeder layers [31].

The labour-intensive nature of T-flasks limits their throughput and applicability to larger-scale processes [18, 25]. Systems that stack multiple culture chambers above one another vertically have enabled greater cell yields than traditional 2D culture methods at lower factory floor footprints [43]. The Cell Factory (ThermoFisher Scientific, Waltham, MA, USA), CellStack/ HyperStack (Corning, New York, NY, USA) and Xpansion (Pall Life Sciences, Port Washington, NY, USA) systems are examples of this. Inflated facility size requirements and subsequent capital investment costs associated with 2D culture scale-up are challenges facing companies hoping to produce hPSCs on a commercial scale [44, 45].

Automated, closed-process systems such as the CompacT SelecT (Sartorius AG, Gottingen, Germany), capable of handling 90 × T175 flasks simultaneously, and the Nunc Automatic Cell Factory Manipulator (ThermoFisher Scientific), capable of manipulating 4 × 40 layer vessels, may help increase the throughput of 2D hPSC bioprocess strategies [46] during expansion and differentiation. Automated systems allow processing to take place in smaller, lower clean rooms compared to manual processing. The closed processing offered by automation systems provides greater process control and reproducibility compared to manual processes [39].

Planar processing platforms will continue to have a place in commercial hPSC bioprocesses. This is particularly likely for the production of autologous cell therapies and patient-specific hPSC-derived cells for personalised medicine drug screening that necessitate a scale-out, rather than a scale-up approach to bioprocess design [43].

### 3.2 Three-dimensional culture systems for hPSC expansion

There are two main methods of 3D hPSC culture; the use of suspended microcarriers as adherent surfaces for stem cell growth [47], or growth of hPSCs as suspended aggregates in SUBs [48]. 3D hPSC culture systems allow online process monitoring, provide greater scalability potential and reduce facility size requirements when compared to planar technologies. Bioreactor systems also permit strict control of conditions during bioprocesses [49]. A challenge to implementation of 3D culture of hPSCs is exposure of cells to shear forces, which must be tightly controlled as they can impact upon hPSC fate determination [50, 51]. Development of xeno-free, defined media [52] is crucial to the robust bioprocessing of hPSCs. The simplicity of such media may reduce costs associated with hPSC expansion materials by 30–60% [53].

#### 3.2.1 Microcarrier-based systems for hPSC expansion

Microcarriers are small beads or discs, which permit propagation or directed differentiation of hPSCs within a 3D bioreactor. hPSC studies investigating microcarriers have recently achieved expansion folds as high as 28-fold over 6 days [54]. Microcarrier-based expansion folds are often higher than those in 2D expansion studies across similar timescales [46, 47, 55–57]. Several recently published reviews include summary tables for SUB-based hPSC expansion studies (using both microcarrier and aggregate culture strategies [25, 28, 29].

A critical property of microcarriers is their high surface area to volume ratio, on which large populations of hPSC cells may be cultured in a relatively small vessel, alleviating the costs associated with expensive media and supplements necessary for hPSC bioprocessing [28, 56]. Microcarriers are able to support expansion and long-term self-renewal of hPSCs over multiple passages, proving the platform's capability to support production of clinically relevant cell numbers [47]. Microcarrier culture of hPSCs also results in cell colonies that generally have less than 10 layers [58]; thus concentration profiles of nutrients and signalling molecules are less likely to occur than in aggregate-based cultures.

The common use of microcarrier coatings that contain animal-derived components, which are unsuitable for use in the manufacture of cell therapies, is a significant challenge to culture of hPSCs on microcarriers. Serum-free and feeder-free microcarrier platforms are available for hPSC-based processing, although many of these utilise the microcarrier coating, Matrigel, which is derived from murine origins [25]. Recombinant human proteins can now be used as a substitute for animal-derived microcarrier coatings in planar and 3D microcarrier cultures [59, 60]. However, such proteins can be difficult to isolate, expensive to produce, and prone to lot-to-lot variation. Synthetic substrates, which circumvent consistency issues associated with recombinant substrates, have been developed in planar conditions and successfully applied to microcarrier-based hPSC culture [61–63].

Xeno-free microcarrier coatings utilise polymers to mimic Matrigel and feeder layer properties in order to encourage attachment and self-renewal of hPSCs on microcarriers. Expansion folds on xeno-free microcarriers comparable to those coated with Matrigel have been reported [60, 63]. It has been proposed that positively charged microcarriers can be used to successfully support hPSC expansion at clinically relevant scales and similar cell concentrations and expansion folds to coated microcarriers were achieved [58]. Methods of xeno-free hPSC culture represent a regulatory compliant approach to the production of hPSCs for clinical applications; they also reduce additional expenses incurred by the use of supplementary serums. Development of xeno-free microcarrier coatings is one area where a quality-by-design (QbD) approach to product development has allowed elucidation of specific properties of microcarriers that affect hPSC self-renewal.

Harvesting of cells from microcarriers is usually carried out using enzymatic separation, which can add to material costs associated with microcarrier-based culture of hPSCs. Microcarriers coated in thermo-sensitive polymers that allow detachment of seeded cells obviate the need for dissociation enzymes, although studies in this area are still in their preliminary phases in this area [64].

Parallel to developing GMP-based hPSC expansion protocols, research has focused on optimising bioreactor conditions for dynamic hPSC expansion processes so as to increase achievable expansion folds and cell concentrations. This will help reduce COG associated with manufacture of hPSCs. Controlling the dissolved oxygen levels has been found to be critical during hPSC culture on microcarriers in SUBs; 2.5 higher expansion folds and ∼85% improvements in maximum cell concentrations were reported in a hypoxic environment when compared to uncontrolled conditions [57]. Furthermore, attachment of hPSCs to microcarriers as single cells can improve seeding efficiency from 30 to over 80% and reduce durations associated with microcarrier loading compared to clump seeding [63].

#### 3.2.2 Aggregate suspension culture of hPSCs

hPSCs can be cultured as suspended aggregates in bioreactors. When hPSCs are grown as aggregates the rho-associated protein kinase inhibitor (ROCKi), Y-27632, is used to protect single cells from dissociation-induced apoptosis [65]. Each cell aggregate is treated as a de facto colony. Aggregate sizes must be controlled in suspension bioreactors to prevent differentiation of cells in larger colonies [66–68]. It has been reported that aggregate culture of stem cells increases the therapeutic potential and the differentiation efficiency of hPSCs via the sustainment of endogenous signalling within cell colonies [32]. Aggregate expansion of hPSCs also negates the need for expensive (and sometimes undefined) components of substrates upon which hPSCs are cultivated in adherent cultures [67]. Aggregate culture of hPSCs rely more heavily on the expensive media supplements (such as GFs) compared to microcarrier culture [54]. Several groups have proposed methods of hPSC culture through the use of cell aggregates with the potential to be scaled up in order to produce clinically relevant cell numbers [67–69]. Twenty-fivefold expansion has been achieved over 14 days during aggregate-based hPSC culture [49] and studies into expansion of hPSCs as aggregates yield similar expansion-folds when compared to microcarrier systems [70–72]. Long-term maintenance of hPSCs in aggregates in dynamic bioreactor conditions over several passages has also been proven to be feasible [49, 68]. Aggregate-based hPSC cultivation necessitates frequent manual interactions in order to control aggregate sizes [28, 71, 73], which will adversely affect labour costs and the robustness of aggregate-based hPSC bioprocesses.

Agitation rates can be used to successfully modulate uniform aggregate size in order to improve expansion of hPSCs as aggregates and reduce cell loss due to shear forces [53, 68]. This is also the case with microcarrier cultures, where impeller speeds of between 45 and 60 rpm were found to promote optimal cell population doubling times [74]. The effects of shear on hPSC self-renewal and lineage determination is an area of intensifying research, although currently this is a poorly understood area in terms of the effect of mechanical strain on hPSC fate determination [29, 75].

The importance of cell inoculation concentration has been demonstrated during aggregate culture of hPSCs in dynamic bioreactor conditions; seeding concentrations of 2–3 × 10^5^ cells/mL were found to maximise viability of hPSCs [68, 76]. Single cell inoculation has also been estimated to reduce cell losses by up to 60% [68]. Cell concentrations of up to 3.4 × 10^6^ hPSCs/mL have been achieved using dynamic, aggregate-based culture techniques [76]. This represents 1.9-fold improvement over maximum cell concentrations achieved in planar systems, although it is significantly lower than the maximum cell concentrations achieved in xeno-free hPSC cultures performed in microcarrier-based systems (6 × 10^6^ cells/mL) [77].

A few aggregate hPSC expansion processes combine xeno-free conditions with defined media [53, 69, 71]. These investigations represent a valuable effort to remove media supplements that either introduce the risk of xenogeneic material to hPSCs or expose media to lot-to-lot variability, although early attempts resulted in relatively modest expansion folds [69].

## 4 hPSC bioprocessing strategies: Differentiation

### 4.1 Planar strategies for hPSC differentiation

Traditional stem cell differentiation protocols were designed around bench-scale research paradigms and little effort was made to incorporate reproducibility and process robustness into these experiments [78]. Directed differentiation strategies often involve exposing hPSCs to a cocktail of morphogens, at specific time-points throughout the differentiation process [79–82]. Similar to hPSC expansion, traditional differentiation strategies rely heavily upon the use of xenogeneic materials [83]. Planar differentiation protocols, that are free of xenogeneic material, have been reported [84]. Despite such progress, many differentiation protocols are inherently variable owing to the laboratory idiosyncrasies of individual technicians, thus reliable and robust differentiation processes are still in their infancy. Timescales and efficiencies also vary significantly between experiments even when the same cell type is targeted for production (Table 3).

**Table 3:** Key performance characteristics of planar and bioreactor-based differentiation protocols

Derived cell-type	Method	Time (days)	Number of target cells per input hPSC (ratio)	Reported efficiency (%)	Max. cell concentration (cells/mL)a)	Refs.
Cardiomyocyte	2D monolayer	9	ND	64.8 ± 3.3	2.5–5 × 10^4^	[87]
Cardiomyocyte	2D EB formation	60	0.81	10 ± 2 – 22 ± 4	ND	[80]
Hepatocytes	2D EB formation	ND	ND	50 ± 2	1–5 × 10^4^	[81]
Hepatocytes	2D monolayer	14	ND	73 ± 18	ND	[82]
Motor neurons	2D monolayer	14	ND	33.6 ± 12	ND	[79]
Neural nociceptors	2D monolayer	15	ND	61 ± 2	1 × 10^4^	[88]
Neurons	2D monolayer	∼7	ND	ND	4.5 × 10^4^	[84]
Dopaminergic neurons	2D monolayer	∼28	ND	30 ± 2	ND	[89]
Neural progenitor cells	2D monolayer	6	ND	90 ± 1	5 × 10^4^	[91]
Endoderm progenitors	2D monolayer	4	ND	73.2 ± 1.6	1.3 × 10^5^	[90]
Cardiomyocytes	2D EB formation	16–18	70	87 ± 3.4	4.5 -6 × 10^4^	[97]
Cardiomyocytes	SUB microcarriers	16	0.33	15.7 ± 3.3	1.36 × 10^6^	[93]
Haematopoietic cells	SUB microcarriers	7	4.41	ND	ND	[92]
Cardiomyocytes	SUB cell aggregates	18	23	100% beating aggregates	4.3 × 10^5^–5.2 × 10^5^	[96]
Hepatocyte-like cells	SUB cell aggregates	21	ND	18 ± 7	3–5 × 10^5^	[94](HLCs)

ND, no data.

a) In planar studies cell concentrations per mL have been estimated based on cell densities and recommended working volume for vessels used in these studies as no cell concentrations are provided in studies of this type.

The use of small molecules within differentiation protocols has helped to improve their reproducibility via reduced use of recombinant growth factors [85, 86]. Several groups have created highly simplified protocols, whilst still improving the efficiency and processing times of 2D differentiation processes, by replacing growth factors with small molecules in the preliminary stages of differentiation protocols [84, 87, 88]. A protocol with the specified aim of creating a differentiation process capable of creating dopaminergic neurons for transplantation in T-flasks has been developed [89]. This strategy enabled the production of cryopreservable dopaminergic neurons with a high level of efficiency, allowing better control of time management in within the differentiation process. This approach is well served to reduce bottlenecks in the downstream phases of autologous hPSC processes. Further advances have proven it is possible to derive progenitor cells, suitable for transplantation as cell therapies at high efficiencies [90] and in xeno-free and small-molecule free, defined conditions [91]. Negation of the need for small molecules and growth factors also has the potential to drastically reduce the cost of differentiation procedures.

### 4.2 Bioreactor-based systems for hPSC differentiation

Concentrated research into bioreactor-based differentiation strategies has stemmed from the need to translate differentiation from a lab-scale area of research into processes capable of producing industrially relevant cell numbers in a reproducible manner. A number of studies in which hPSCs have been successfully differentiated in bioreactor conditions have been carried out, either attached to microcarriers [92] or in the form of cell aggregates [93, 94]. Differentiation strategies developed in 3D bioreactors, particularly stirred-tank vessels, lend themselves to large-scale processes far better than their planar counterparts as labour-intensive tasks, such as media exchanges, can be fully automated in such vessels, which also offer processing advantages such as online environmental monitoring and control. Research carried out on SUB-based differentiation of mPSCs suggests that the use of spinner flasks resulted in a 12-fold reduction of the man hours spent in the laboratory when compared to planar techniques [95].

hPSCs can be differentiated towards a number of clinically relevant lineages in SUBs including cardiac [96], haematopoietic [92], neuronal [77] and hepatocyte-like [94] (see Table 3 for summary). Bioreactor-based differentiation will be necessary in order to produce certain hPSC-derived cell products at commercially relevant scales, however it must be considered that such processes will only be made more cost-effective by making concurrent improvements in differentiation efficiencies and through the reduction of expensive media supplements in such protocols. Bioreactor-based differentiation would benefit from the translation of highly efficient protocols demonstrated in planar systems [88, 91, 97] to SUB systems. Such protocols have the potential to result in differentiation efficiencies that are higher than those achieved with SUB-based bioreactors alone. A novel, microparticle-based approach to morphogen delivery to PSC aggregates was reported as a method to achieve up to a 12-fold reduction in morphogen use during bioreactor-based differentiation protocols [98]. Such systems provide a valuable method by which to reduce material costs associated with SUB-based hPSC culture.

One question arising from the birth of SUB-based hPSC differentiation is how a dynamic, controlled environment might be harnessed to augment processing strategies in this area [51]. One of the reasons for the lack of characterisation with regards to the effects of shear on hPSCs, is that different dynamic culture systems result in different shear profiles. Thus, drawing comparisons across separate studies is difficult [75]. However, scale-down studies suggest that shear stress during early hPSC differentiation promotes mesodermal, endothelial and haematopoietic phenotypes even when the presence of morphogens promoting these lineages were absent [99–101]. Interestingly, in early stages of differentiation, hPSCs lineage determination seems to be insensitive to the magnitude of shear stress; however in later stages, progenitor cell activity appears to be more magnitude-sensitive [100, 102]. Shear forces have been shown to partially negate the need for costly media supplements in published studies [99, 100]; broadening our knowledge of the way that shear stress impacts upon hPSC culture must be seen as an important factor in bioprocess optimisation. Hypoxic environments, which can be tightly controlled within SUBs, have also been shown to enhance differentiation of hPSCs towards both ectoderm and mesoderm cell lineages [96, 103].

Novel, microfluidic systems could be a key tool in elucidating the effects of specific environmental parameters on hPSC propagation and differentiation. Microfluidic bioreactors provide an ultra-scale down, high throughput platform by which to study single cells or colonies in strictly defined conditions [104–107]. The cellular processes governing hPSC fate are complex and cannot be attributed to a single given parameter. Microfluidic devices are well placed, as a low cost development platform, to enhance our understanding of how defined microenvironmental conditions can affect hPSC activity [108].

The effect of the biochemical properties of microcarriers on hPSC fate determination is poorly understood. It has also been suggested that the mechanical properties of microcarriers, such as their stiffness and size can also be investigated and optimised for specific purposes [27]. Rational design of microcarriers could provide an optimized bioprocess platform with which to manufacture specific hPSC-derived cell lineages. This would enable better control of cells’ microenvironment and thus allow more efficient differentiation processes that do not rely as heavily on expensive media supplements as current platforms do.

## 5 Integrated bioprocess design strategies for hPSCs

**Figure 1:**
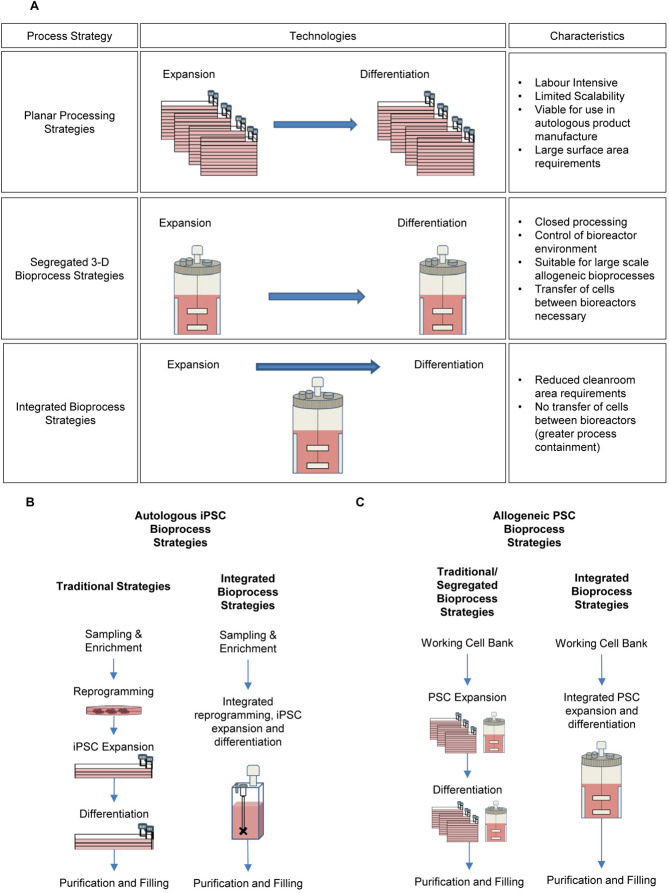
hPSC bioprocess strategies and their characteristics: (A) Planar processing strategies (top panels) rely on multi-layer vessels. Development of SUBs for PSC culture and differentiation has allowed development of 3D strategies, which are suitable for large-scale allogeneic bioprocesses (middle panels). Integrated bioprocesses allow hPSC expansion and differentiation to be carried out as a single unit operation in the same bioreactor (bottom panels). (B) Traditional and integrated autologous iPSC bioprocess strategies: Tradtional planar strategies neccessitate the need for use of well-plates and T-Flasks. Novel, automated bioreactor systems such as the Ambr system (Sartorius AG) may allow implementation of bioprocess strategies whereby reprogramming, iPSC expansion and differentiation are carried out within a single bioreactor. (C) Segregated and integrated allogeneic bioprocess strategies: Tradtional allogneic bioprocess strategies utilise multi-layer, planar technologies. Segregated bioprocessing stratagies make use of separate SUBs for hPSC expansion and differentiation. Integrated bioprocessing allows hPSC expansion and differentiation to be carried out within a single bioreactor as a single unit opearation.

Novel, integrated hPSC bioprocesses, whereby multiple unit operations are carried out using continuous culture strategies, are being explored as an alternative to segregated bioprocess strategies. Integrated bioprocesses negate the need for labour-intensive processes that usually take place following hPSC expansion such as harvest and transfer of cells prior to differentiation. Processing hPSCs in this manner can help to avoid process bottlenecks and increase throughputs of hPSC product manufacture. Additionally, integrated iPSC bioprocess protocols offer greater containment capabilities, reducing the potential for contamination within the bioprocess. Several studies in which hPSC expansion and differentiation are carried out as a single unit operation using continuous culture strategies exist [67, 74, 77]. Figure 1A illustrates how these strategies differ from segregated culture and differentiation of hPSCs. Only a handful of investigations into this relatively new area of hPSC bioprocess research have been published and these are summarised in Table 4.

**Table 4:** Key performance characteristics of studies investigating the integrated expansion and differentiation of hPSCs

Culture conditions	Cell type (iPSC/ESC)	Target cell type	Expansion max.cell density (cells/mL) (fold expansion)	Differentiation max. cell density (cells/mL) (fold expansion)	Reported differentiation efficiency (%)	Process time (days)	Target cells produced per input hPSC	Xeno-free (Y/N)	Refs.
Microcarrier (DE53 Whatman)	hiPSC	Neural progenitor cells (NPCs)	6.1 × 10^6^ (20)	1.1 × 10^6^ (16.6)	78 ± 4.7	25	333	N	[77]
Microcarrier (DE53 Whatman)	hESC	NPCs	4.3 × 10^6^ (21.3)	1 × 10^6^ (17.7)	83 ± 8.5	23	371	N	[77]
Aggregate culture	hESC	Definitive endoderm progenitors (DEPs)	ND (5000)a)	ND (23.5)	> 80%	22	65, 000a)	N	[67]
Microcarrier (collagen-coated hyclone)	hESC	DEPs	1 × 10^6^ (34–45)	4 × 10^5^ (ND)	84.2 ± 2.3%	12	4	N	[74]

Parameters given for both expansion and differentiation where available. ND = No data given.

a) hESCs underwent four rounds of expansion during this study, as opposed to one round of expansion in other studies shown here. This may contribute to the disparity in performance parameters between this study and others shown here. ((Please start this sentence as a new line))

Early investigations incorporating expansion and differentiation focused on overcoming the technical hurdles of carrying out two different operations in one integrated process step; as such only modest yields of target cells were achieved [74]. Recent studies have sought to optimise integrated iPSC bioprocesses via strategies such as the determination of optimal aggregate size during hPSC culture and differentiation [67]. Switching feeding regimes from once to twice per day was found to double the achievable cell density during the expansion phase of an integrated bioprocess, although process economic analysis comparing the two approaches was not offered [77]. The reported expansion folds and differentiation efficiencies for integrated bioprocesses compare well with separated systems of a similar nature (Table 4).

To date, no studies have produced an integrated process for hPSCs from derivation all the way through to differentiation. This has been achieved with mouse iPSCs (miPSCs) in a SUB [109]. To our knowledge, there are only two studies exhibiting ‘suspension culture reprogrammed iPSCs’, both of which deal with miPSCs [109, 110]. Translation of integrated miPSC production techniques described by Baptista et al. [110] to hiPSC processing may be particularly useful in the production of autologous stem cell therapies, where continuous derivation of large numbers hiPSCs would help reduce the bottlenecks bought about by cellular reprogramming. Moreover, it may provide a ‘black box’ platform to derive, expand and differentiate a patient's cells in a single, contained unit that could be installed at point-of-care centres for relevant disease types. Novel, controlled miniature bioreactor systems, such as the ambr15 (Sartorius AG), might offer a suitable platform for autologous hiPSC product manufacture, where limited cell numbers are required and scale-out strategies necessitate alternatives to large-scale bioreactors (Fig. 1B and 1C).

## 6 Concluding remarks

The fulfilment of the clinical potential of hPSCs depends on the development of scalable, robust, GMP-compliant bioprocesses. Concentrated efforts to develop rigorously designed bioprocesses with a greater emphasis placed on QbD will continue to enhance process understanding with respects to defining critical quality attributes and identifying key process variables that must be controlled. hPSC-derived products are subject to strict economic boundaries owing to the nature of global healthcare systems. Future work must build upon the promise of works reviewed in this paper in order to make delivering such products on budget an achievable goal. Novel approaches discussed in this review offer methods to further optimise hPSC bioprocessing platforms.

**Michael J. Jenkins** is a doctoral research student in Biochemical Engineering at University College London (UCL) in the UK. Michael's project, in collaboration with Neusentis Ltd., focuses on cost-effective bioprocess design for hPSC-derived cell products including cell therapies and drug screening tools. His research involves the creation of novel decisional tools that combine process economics analysis, bioprocess optimisation and uncertainty analyses of hPSC bioprocesses. He obtained his Master's degree in Biochemical Engineering at UCL, with a year as an exchange student within the Biotechnology Department at Lund University, Sweden.

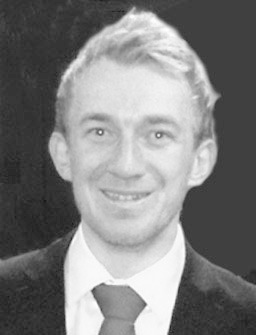

**Suzanne S. Farid** is Professor in Bioprocess Systems Engineering at the Advanced Centre for Biochemical Engineering at University College London (UCL) in the UK and Co-Director of the EPSRC Centre for Innovative Manufacturing in Emergent Macromolecular Therapies. She leads research on ‘Bioprocess Decisional Tools’ that has pioneered the development of algorithms at the process–business interface to facilitate cost-effective bioprocess design, capacity planning and R&D portfolio management. She sits on the UK BioIndustry Association Manufacturing Advisory Committee and is a Fellow of the IChemE. She obtained her Bachelor's and Ph.D. degrees in Biochemical Engineering with UCL and Lonza Biologics.

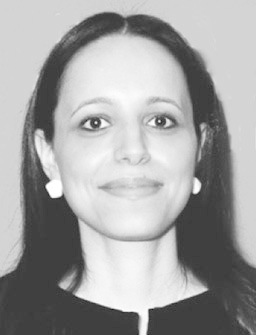

## Abbreviations

COG: cost of goods
cGMP: current good manufacturing practice
cGTP: current good tissue practice
FCI: fixed capital investment
hPSC: human pluripotent stem cell
QbD: quality by design
SUB: single-use bioreactor
